# Removal of COD, aromaticity and color of a pretreated chemical producing industrial wastewater: a comparison between adsorption, ozonation, and advanced oxidation processes

**DOI:** 10.3906/kim-2010-48

**Published:** 2021-06-30

**Authors:** Elçin GÜNEŞ, Deniz İzlen ÇİFÇİ, Ali Rıza DİNÇER, Yalçın GÜNEŞ

**Affiliations:** 1 Department of Environmental Engineering, Çorlu Engineering Faculty, Namık Kemal University, Tekirdağ Turkey

**Keywords:** UV-vis absorbance values, aromaticity, advanced oxidation processes, wastewater treatment, adsorption

## Abstract

A wide range of products are produced in the chemical producing industry such as textile dyes, chemicals, printing dyes and chemicals, paper chemicals, electrostatic powder dyes, and optical brighteners. The aim of this study is to investigate the treatability of chemical oxygen demand (COD), aromaticity, and color in the wastewater of this sector, where highly complex chemicals are used. Most of the studies in the literature are related to the treatment of synthetically prepared dyed wastewater. This study is important as it is carried out with real wastewater and gives results of many treatment methods. In the study, COD, UV-vis absorbance, and color values were attempted to be removed from the wastewater of a chemical producing industry that was pretreated by coagulation-flocculation. The COD value of the pretreated wastewater discharged to the central treatment system was restricted as 1000 mg/L. Pretreated wastewater characterization is as follows: COD: 2117 mg/L, UV-vis absorbance values at; 254 nm: 9.91, 280 nm: 8.65, 341 nm: 12.77, 436 nm: 5.01, 525 nm: 2.24, and 620 nm: 1.59. In the study, adsorption, ozonation, and advanced oxidation processes (Fenton and persulfate oxidation) were used to remove COD and UV-vis absorbance values (aromaticity, organics, and color). The method by which the best removal efficiency was obtained for all parameters was the adsorption process using powdered activated carbon (PAC). The equilibrium PAC dose was found as 6 g/L. At this adsorbent dose, the removal efficiencies of UV-vis absorbance values were all around 99% and the efficiency of COD removal was 77%. The Langmuir isotherm constants were found to be q_max_= 30.4 mg/g and K_L _= 487.9 (L/mg). The COD concentration at this adsorbent dose was 486 mg/L and wastewater was suitable for discharge to the central wastewater treatment plant in that region.

## 1. Introduction

Chemical producing industries are regarded as a highly polluting sector because they produce a wide variety of products. The chemical industry produces a wide variety of products such as paints, pigments, adhesives, sealants, catalysts, and coatings, as well as pharmaceuticals, pesticides, soaps, detergents, shampoos, and creams from various raw materials [1]. The chemical industry generally prefers to deal with the end of the pipe treatment for the management of wastewater. Since chemical producing industries have a wide range of products, wastewater can also contain a large number of refractory and toxic substances. The presence of toxic or inhibitory substances in the wastewater of the chemical producing industries makes it difficult to treat the wastewater originating from this sector. In such cases, various treatment methods are tried, such as adsorption and/or advanced oxidation processes, in addition to the coagulation-flocculation process or activated sludge treatment [2].

Adsorption is an effective physicochemical alternative method used to remove dyes and organics from wastewaters [3]. The adsorption process has many advantages. It is not very sensitive to the daily changes of wastewater quality and while biological treatment is sensitive to toxic chemical, adsorption is not. It is also very flexible in design and operation, and is successful in removing many organic pollutants [3,4]. The adsorption process is widely used by many researchers for the removal of various chemicals, dyes, and many organic substances [5,6]. In the adsorption process, most of the dyes are adsorbed on the adsorbent and do not degrade [7]. The most commonly used material for adsorption is commercial activated carbon [8].

Advanced oxidation processes (AOPs) are techniques that use hydroxyl or sulfate radicals produced on site to remove organic pollutants present in water and wastewater [9]. AOPs are an alternative technology applied for removal of dyes containing wastewater and other organic pollutants and performing well in terms of organics and color removal [10]. There are a wide variety of advanced oxidation processes such as, Fenton, persulfate oxidation, peroxymonosulfate oxidation, UV/H_2_O_2_, and O_3_/H_2_O_2_. The Fenton process is one of the common treatment methods of organic pollutants, dyes, and various toxic substances. Among the different AOPs, Fenton treatment is one of the strongest oxidative treatments operated at room temperature and pressure [5]. Hydroxyl radical with oxidation potential of between 1.9 and 2.85 V is produced by using H_2_O_2_ and Fe^2+^ in the Fenton process. This process is a combination of oxidation, coagulation, and sedimentation [11]. The basic reaction equations providing the OH radical (OH^•^) formation of the Fenton process are given in Eqs. (1) and (2) [12].

Another of the AOPs is sulfate radical-based oxidation. Peroxydisulfate (persulfate) was used in this study for the production of sulfate radicals. Persulfate (S_2_O_8_^2–^) is an oxidant used in chemical oxidation for water, wastewater and soil cleanup and a strong oxidant [13]. Persulfate oxidation takes advantage of the high redox potential of sulfate radicals (SO_4_^•–^) (which has standard oxidation potential between 2.5 and 3.1 V) produced by breaking the O-O bond, either chemically or thermally [14–17]. Production of sulfate radicals from persulfate can take various forms: heat, UV radiation, and transition metals [13,14]. Thermally activated persulfate is considered a clean source of sulfate radicals and has been used in the literature to remove a large number of organic pollutants in many studies [13–18].

Ozonation is also one of the important and widely used oxidation processes. Ozonation is a chemical water and wastewater treatment technique based on the oxidation potential of ozone. Ozone, which is one of the strongest oxidants, is a gas consisting of three oxygen atoms (O_3_), ozone has a high oxidation potential (2.07 V), even higher than those of chlorine (1.36 V) and H_2_O_2_ (1.77 V) [19]. Ozone oxidation is one of the advanced treatment processes used in the treatment of wastewaters. When the ozonation process is applied under favorable conditions, hydroxyl radicals formed in water quickly break down the double bonds in the structure of the dyes and provide high organic matter and color removal [20]. 

In this study, the treatment of a chemical producing industrial wastewater that was pretreated using the coagulation-flocculation (with FeCl_3_) process, with adsorption and advanced oxidation processes was investigated. In the chemical producing industry products such as textile dyes, textile chemicals, mineral oils (textile lubricating oils, blending oils, and transfer oils) are produced. In order to add value to polyester, cotton, acrylic and polyamide fiber, disperse dyes, reactive dyes, acrylic dyes, acid dyes, pigments, and chemical groups offer products in various forms as liquid, powder, and disperse. In addition, it produces hybrid electrostatic powder dyes for metal industry, and paper auxiliaries, brown paint, optical brightener, and performance chemicals for paper industry. The wastewaters of this industry contain quite complex substances depending on the product variety it produces. In addition, it is quite problematic in terms of organic matter, aromaticity, and color removal from wastewater. Most of the studies in the literature are about the removal of color and organic matter from wastewaters prepared with synthetic dye mixtures. However, in addition to color and organic substances, there are a wide variety of salts, auxiliary chemicals, various dyes, and dangerous chemicals in real wastewaters. What makes this study original is that research has been conducted with real wastewater containing highly complex chemicals and four different treatment methods were used to remove organics, aromatics, and color. Moreover, various treatment methods were compared. In this study, removal efficiencies of COD and removal efficiencies of absorbance values (UV 254, 280, 341, 436, 525, and 620 nm wavelengths) were evaluated on wastewater which was pretreated by coagulation-flocculation of a chemical producing industry. As it is known, absorbance at 254 nm wavelength is typical for measurements of aromatic compounds (aromaticity) [21]. The most common wavelengths for natural organic materials measurements are from 220 nm to 280 nm [21,22]. The removal efficiency of absorbance at UV 341 nm was also measured in the study because the maximum absorbance of wastewater was measured at this wavelength. Typically, the human eye can detect wavelengths from 400 nm to 700 nm (visible light). Color can be measured by absorbance measurements between these wavelengths. In this study, absorbance values at 3 different wavelengths (436, 525, and 620 nm) were measured to measure color removal of this wastewater. In the study, UV-vis absorbance values (aromaticity and color) at 254–620 nm wavelengths and COD removal efficiencies were investigated using the adsorption method, Fenton process (this process includes oxidation-coagulation and sedimentation together), persulfate oxidation, and ozonation processes.

## 2. Materials and methods

### 2.1. Wastewater characterization

Wastewater was collected from a chemical industry which produce disperse dye, reactive dye, acrylic dye, acid dye, pigments, and chemical groups to add value to polyester, cotton, acrylic, and polyamide fibers. Wastewater from this chemical industry which is located in Çerkezköy, Tekirdağ, Turkey, is pretreated by the coagulation-flocculation process using FeCl_3_ as a coagulant and then discharged to the centralized wastewater treatment facility. It is required that the COD value of wastewater is below 1000 mg/L to discharge wastewater to network of centralized wastewater treatment facility. For characterization and treatment experiments, the wastewater sample was collected in 20 L plastic bottles, immediately transported to the laboratory, and preserved and stored according to standard methods [23]. 

The characterization of the wastewater is given in Table 1. As shown in Table 1, COD is higher than the discharge standard required for the centralized wastewater treatment facility. The total suspended solids (TSS) concentration in the wastewater is quite low which shows the particulate matter in the wastewater. The wastewater has a brown-red color. The absorbance values between 400 and 700 nm wavelengths, which are the measure of color, were measured using UV 436–525 nm and 620 nm wavelengths in the study. As it is seen from the table, the absorbance values at UV436-UV525-UV620 nm wavelengths in the wastewater are quite high. According to Szabo and Tuhkanen (2016), the area under the UV-vis spectra between 250 and 350 nm and A254 (absorbance at 254 nm) are applicable surrogates of COD and DOC (dissolved organic carbon) [24]. UV254 and UV280 values are a measurement of the amount of light absorbed by organic compounds, specifically aromatics, in a water sample [17,20]. As shown in Table 1, the absorbance values of UV254 nm and UV280 nm wavelengths are 9.91 and 8.65, respectively. The maximum absorbance value of the wastewater was measured at 341 nm wavelength. As shown in Table 1, the absorbance at this wavelength is very high and measured as 12.77 abs. 

**Table 1:** The characterization of the wastewater studied.

Parameter	Unit	Value
pH	-	8.08
EC	mS/cm	6.78
TSS	mg/L	18
VSS	mg/L	12
NH3-N	mg/L	17.36
TKN	mg/L	66.64
COD	mg/L	2117
Dissolved COD	mg/L	2061
UV-vis absorbance values		
254 nm	abs	9.91
280 nm	abs	8.65
341 nm	abs	12.77
436 nm	abs	5.01
525 nm	abs	2.24
620 nm	abs	1.59

### 2.2. Treatment studies

#### 2.2.1. Adsorption process

In the adsorption experiments, powdered activated carbon (PAC-Norit SA 2, Acros Organics, which has 686 m^2^/g specific surface area) was used as the adsorbent. As a result of experiments to determine the point of zero charge pH (pHpzc) of activated carbon, pHpzc was found as 7.45. In the adsorption study, as a first step, optimum pH was determined. After the optimum pH value was determined, the optimum dosage was determined by changing the activated carbon dosages. Activated carbon dosages of 0.1, 0.15, 0.2, 0.25, 0.3 g were placed in 50 mL of wastewater and shaken at 200 rpm for 2 h. After 2 h, the samples were taken and centrifuged at 4000 rpm for 15 min, and then the samples were taken from the supernatant to determine COD, UV 254–280 nm (organics and aromatics), UV 341 nm (wavelength at which maximum absorbance is measured), and 436, 525, 620 nm (color).

#### 2.2.2. Advanced oxidation processes

##### 2.2.2.1. Fenton process

For the Fenton experiments H_2_O_2_ (35% w/w) FeSO_4_·7H_2_O, NaOH and H_2_SO_4_ (all purchased from Merck, Darmstadt, Germany) were used. Fenton processes were conducted by using the jar test method at room temperature and at a constant pH (pH 3) using varying H_2_O_2_ and FeSO_4_·7H_2_O dosages to determine the optimum conditions for the best COD, color, and aromaticity removal. H_2_O_2_ dosages were 1000, 2000, 3000, 4000, and 5000 mg/L and Fe^2+^ dosages were 400, 600, 700, 800, and 900 mg/L. Firstly, each beaker was filled with 100 mL of the wastewater, and the pH was adjusted to 3 with one NaH_2_SO_4_ solution. Then FeSO_4_·7H_2_O and H_2_O_2_ were added in required amounts into the wastewater. For the determination of optimum H_2_O_2_ dosage in COD, color and aromaticity removal efficiency, Fe^2+^ dosages were kept constant at 500 mg/L. Then at this Fe^2+ ^dosage, 1000–5000 mg/L H_2_O_2_ was added to the samples and optimum H_2_O_2_ dosage was determined. At optimum H_2_O_2_ condition between 400 and 900 mg/L Fe^2+^ dosages were added to the samples and optimum Fe^2+^ dosage was determined. All samples were stirred in a Jar-test apparatus for 10 min at 120 rpm, and for 60 min at 60 rpm. Then the pH was adjusted to 7.5–8.5 with 1 N NaOH to precipitate Fe(OH)_3 _and to decompose H_2_O_2_ to turn into O_2_ and H_2_O, as well as to prevent interferences of H_2_O_2_ to COD test [25]. After waiting for 1 h, the supernatant was withdrawn for COD, UV254-UV280-UV341-UV436-UV525-UV620 nm absorbance analyses.

##### 2.2.2.2. Persulfate oxidation 

The thermal activation method was used to produce the sulfate radical (SO_4_^•−^) from persulfate (K_2_S_2_O_8_)(Merck). A magnetic stirrer with a contact thermometer was used to adjust the operating temperatures (IKA-C MAGHS7-480). In this study, 5 different persulfate doses were used depending on the COD/S_2_O_8_ ratio. Persulfate doses were added as 1/1, 1/1.5, 1/2, 1/2.5, and 1/5 ratios depending on the COD/S_2_O_8_ ratio [26]. In this study, no studies on the effect of pH change have been made. In order to determine the effect of the oxidation time, samples were taken every half hour: 0.5, 1, 1.5, 2, 2.5, 3, 3.5, 4, 4.5, 5, and 5.5 h. In order to determine the effect of temperature, 3 different temperatures were tested: 60, 65, and 70 °C. After each contact time, samples were taken and the oxidation reaction was stopped by putting them in ice water immediately. The contact time was taken as 5.5 h considering the time to reach equilibrium. When the samples were taken, the water level was measured on the beaker and the evaporation loss was prevented by the addition of distilled water.

#### 2.2.3. Ozone oxidation

An ozone generator manufactured by Degremont with a production rate of 1 g O_3_ per hour was used to supply ozone. The ozonation system was operated in a semicontinuous type, i.e. continuous with respect to the gas flow and batch with respect to wastewater. Two liters of wastewater was filled into a 4 L stainless steel reactor. Samples were taken at 0.5, 1, 1.5, 2, 2.5, 3, 3.5, 4, 4.5, 5, and 5.5 h to compare the results with persulfate oxidation results. Excess ozone gas passed out through the top of the reactor into a gas-washing bottle containing KI solution. The amount of ozone in the effluent gas was measured by taking samples from the KI trap during experimental run and titrating the iodine in the samples with Na_2_S_2_O_3_ according to standard methods [23]. Two gas-washing bottles containing KI solution were connected to the system in parallel with the reactor to determine the quantity of ozone applied to the reactor.

### 2.3. Analysis

The pH was measured using a pH meter (WTW pH 315i). Total COD, dissolved COD, total suspended solids (TSS), volatile suspended solids (VSS), total Kjeldahl nitrogen (TKN), and ammonia nitrogen (NH_3_-N) were analyzed according to standard methods [23]. COD was determined using a closed reflux colorimetric method. The S_2_O_8_ concentration was measured according to Liang et al. [27]. 

As it is known, UV254 is used for aromatic and unsaturated organic compounds and UV280 is represent aromaticity [28]. Color (UV436-UV525-UV620), organics, aromaticity (UV254-UV280), and wavelength at which maximum absorbance of the wastewater is detected (UV341) were measured using a UV-vis spectrophotometer (Shimadzu UV-2401 PC instrument, Kyoto, Japan). The absorbance values of the turbid samples were pretreated by centrifugation at 3500 rpm for 5 min and measured using the UV-vis spectrophotometer. The dilution technique was done for all samples before measuring the absorbance of the solution to confirm the Lambert–Beer law. 

## 3. Results

### 3.1. Adsorption process

#### 3.1.1. Effect of pH 

**Figure 1:**
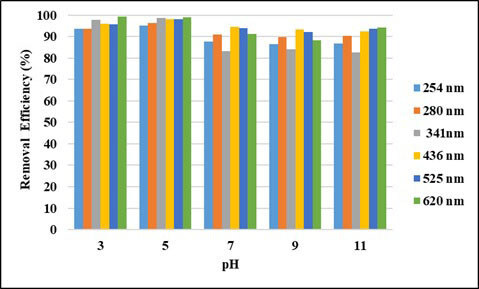
pH effect on UV-vis absorbance values removal efficiencies (PAC dose: 4 g/L, contact time: 2 h).

**Figure 2:**
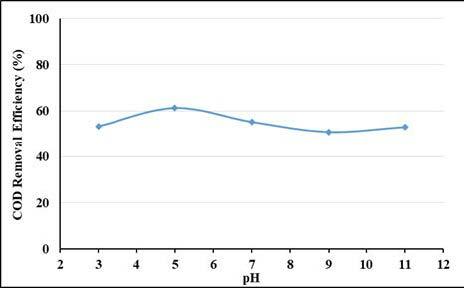
pH effect on COD removal efficiencies (PAC: 4 g/L, contact time: 2 h).

pH is one of the most important parameters affecting the removal efficiency in adsorption process. The adsorption capacity and removal efficiencies of pollutants are affected as the surface charge of the adsorbent changes with pH change. The removal efficiencies of UV-VIS absorbance values and COD by powdered activated carbon (PAC) at different pHs are shown in Figures 1 and 2. As can be seen from the figures, the removal efficiencies of COD and UV-vis absorbance values were higher at low pH. The highest removal efficiencies were reached at pH: 5 and COD removal efficiency was highest with 61.2% at this pH. It has been determined that the pHpzc of activated carbon is 7.45. Accordingly, when the pH is below 7.45, surface of activated carbon is positively charged and tends to attract negatively charged pollutants. When the effects of pH on the removal efficiencies of UV-vis absorbance values are evaluated, it can be said that the surface of pollutants in this wastewater, which contains a wide range of dyes and organics, are negatively charged under pH: 7.45, so they are highly absorbed by the adsorbent. It is estimated that the removal efficiency decreases due to the deterioration of the structure of activated carbon or pollutants below pH: 5. Moreover, it is estimated that at an acidic pH the presence of excess of H^+^ ions compete with the wastewater components for the adsorption sites [29].

#### 3.1.2. Effect of adsorbent dosages

**Figure 3:**
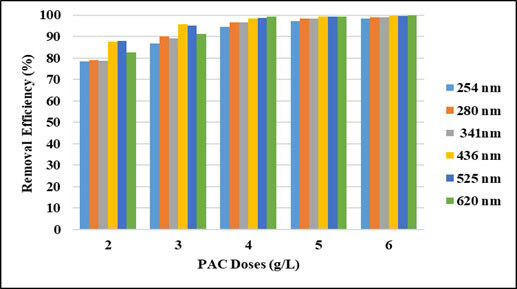
Effect of adsorbent doses on UV-vis absorbance values removal efficiencies.

**Figure 4:**
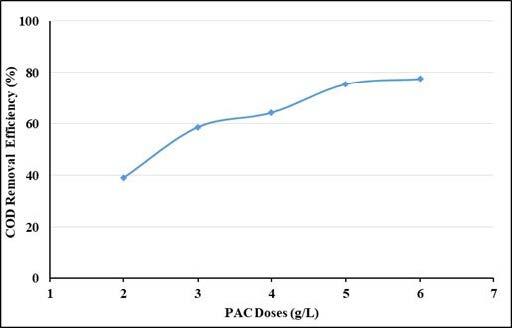
Effect of adsorbent doses on COD removal efficiencies.

The effect of the PAC dosages on the removal efficiencies of UV-vis absorbance values and COD were evaluated at pH: 5 of the wastewater with a constant mixing rate of 200 rpm. Test results are shown in Figures 3 and 4. As seen in the figures, as adsorbent doses increase, UV-vis absorbance values removal efficiencies and COD removal efficiencies increase, as expected, and then reach equilibrium. The removal efficiencies increased as the adsorbent doses increased from 2 to 6 g/L. As shown in Figure 3, the equilibrium dose of PAC was found to be 6 g/L. In this adsorbent dose, UV-vis absorbance values removal efficiencies were around 99% and COD removal efficiency was 77%. The COD concentration in this adsorbent dosage decreased to 486 mg/L and was suitable for discharge to the centralized wastewater treatment facility.

#### 3.1.3. Isotherm studies

**Figure 5:**
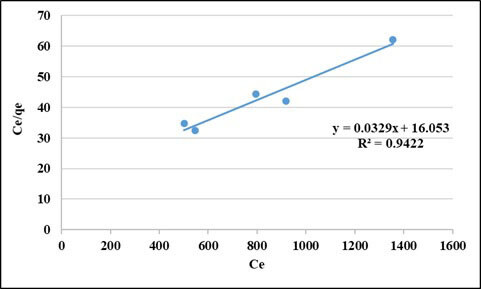
Langmuir isotherm curve for COD removal (pH: 5, t: 2 h, PAC doses: 2–6 g/L).

**Figure 6:**
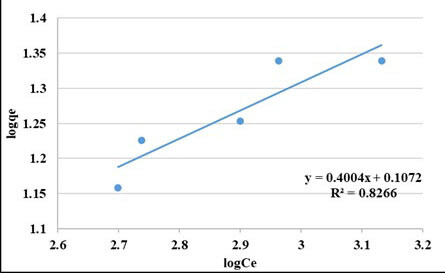
Freundlich isotherm curve for COD removal (pH: 5, t: 2 h, PAC doses: 2–6) g/L).

The equilibrium characteristics of adsorption were described by different isotherm models in the literature. In this study, the most widely used isotherms models, Langmuir and Freundlich models were employed. Langmuir and Freundlich isotherm plots are presented in Figures 5 and 6, respectively. As can be seen from the figures, R^2^ values for Langmuir and Freundlich isotherms are 94% and 83%, respectively. It can be said that Langmuir model yields a better fit than the Freundlich model for the adsorption of COD of this wastewater on the PAC used this study. The Langmuir isotherm constants were found to be q_max _(the amount of adsorbate required to form a monolayer) = 30.4 mg/g and K_L_ (the equilibrium constant) = 487.9 (L/mg) [29].

Freundlich isotherm constants were found as K_f_ = 2.7 mg/g and 1/n = 0.4. Values of 1/n<1 show the favorable nature of adsorption of COD on the PAC used in this study [30].

### 3.2. Advanced oxidation processes

3.2.1. Fenton process 

Many studies have shown that the Fenton process’s pollutant removal efficiencies are optimum at pH: 3. Therefore, in this study, optimum pH experiments were not conducted, and Fenton experiments were done at pH: 3. In the study, optimum H_2_O_2_ dosage was determined first, and then optimum Fe^2+^ dosage was determined by changing Fe^2+^ doses at optimum H_2_O_2_ dosage.

The process of Fenton is based on the production of hydroxyl radicals by the decomposition of hydrogen peroxide catalyzed by iron ions [31]. The favorable effect of H_2_O_2_ addition on reaction kinetics is generally associated with generation of a higher OH^•^ quantity. However, this effect occurs to a certain H_2_O_2 _concentration. Above this limit, an excess of OH^•^ occurs and the OH^•^ radicals react with H_2_O_2_ and can cause formation of hydroperoxyl radicals (Eq. (3)). Since the hydroperoxyl radicals have less oxidation power than OH^•^, the reaction efficiency will be reduced. In addition, also excess Fe^2+^ dosages in Fenton process reduce the removal efficiency of pollutants. The following equations show why removal efficiencies decrease when H_2_O_2_ and Fe^2+^ concentrations are excessive (Eqs. (3)–(5)) [32–33]. Excess H_2_O_2_ forms hydroxypyroxyl radicals which has low oxidation power, and excess Fe^2+^ has scavenging effect for OH^•^.

**Figure 7:**
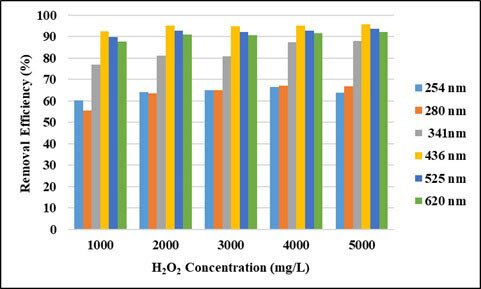
Effect of H2O2 concentration on removal efficiencies of UV-vis absorbance values (pH: 3, Fe2+: 500 mg/L).

**Figure 8:**
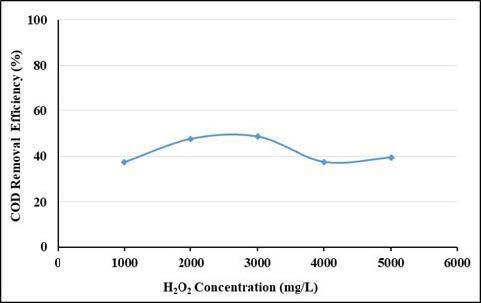
Effect of H2O2 concentration on COD removal efficiencies (pH: 3, Fe2+: 500 mg/L).

In this study, initially Fe^2+^ dosage were fixed at 500 mg/L and the dosage of H_2_O_2_ increased from 1000 mg/L to 5000 mg/L. Figures 7 and 8 show removal efficiencies of UV-vis absorbance values and COD removal efficiencies in the range of 1000–5000 mg/L H_2_O_2 _concentration. As seen in Figure 7, removal efficiencies of UV-vis absorbance values did not change much with H_2_O_2_ concentrations. Even at the lowest H_2_O_2_ doses, the absorbance removal efficiencies of UV 436, 525, 620 nm wavelengths were above 85%. When the COD removal efficiencies are examined, it is seen that the highest removal efficiency is at 3000 mg/L H_2_O_2_ doses and the removal efficiency is up to 48.6% at this dose. The COD removal efficiency started to decrease for doses of H_2_O_2 _over 3000 mg/L. This may be explained by the scavenging activity of OH• by reacting with H_2_O_2_ [34]. The optimum H_2_O_2_ concentration was determined to be 3000 mg/L. 

It is stated in the literature that organic parameters such as COD, TOC, DOC, and BOD show maximum absorbance at wavelengths between UV 250–380 nm [28]. When the results in this study are examined, it is seen that the removal efficiencies of absorbance values at UV 254 and UV 280 nm wavelengths remain at 60% and are relatively compatible with COD removal efficiencies.

**Figure 9:**
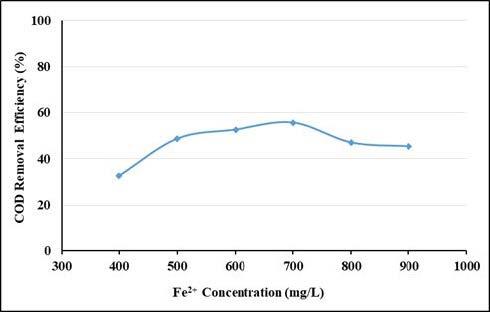
Effect of Fe2+ concentration on removal efficiencies of UV-vis absorbance values (pH: 3, H2O2: 3000 mg/L).

**Figure 10:**
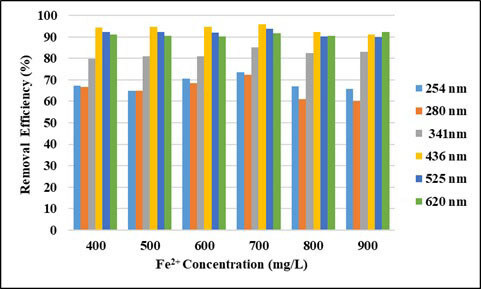
Effect of Fe2+ concentration on COD removal efficiencies (pH: 3, H2O2: 3000 mg/L).

Figures 9 and 10 show the effect of Fe^2+^ dosage on the UV-vis absorbance values and COD removal efficiencies with a fixed dose of 3000 mg/L H_2_O_2_ for the wastewater. When the UV-vis absorbance values removal efficiencies (Figure 9) are examined, it is seen that the efficiency at 400–900 mg/L Fe^2+^ doses are almost constant. The absorbance values removal efficiencies at UV254 and UV280 nm wavelengths vary between 65% and 73%. It is seen that the absorbance removal efficiencies at UV341 nm wavelength vary between 79% and 85%. The absorbance removal efficiencies at the wavelengths of UV 436–525–620 nm are quite high and are above 90%. As seen from Figure 10, as the dosage of Fe^2+^ increased from 400 to 700 mg/L the COD removal efficiency increased from 32.5% to 55.6%. COD removal efficiency decreased at Fe^2+^ doses above 700 mg/L. In many studies, the efficiency of Fenton reaction decreases in Fe^2+ ^concentrations above a certain value [32]. This relates to the scavenging effects of increased Fe^2+^ on OH^•^ as seen in Eq. (4) [33].

As it is known, the groups that give color to organic substances are chromophore groups. These substances give the true color of the organic matter and are measured between UV 400 and 700 nm. When the study results are examined, it is seen that the removal efficiencies of UV 400–700 nm wavelengths are quite high. According to these results, it can be said that chromophore groups can be easily broken down by oxidation and therefore their removal efficiency is very high.

#### 3.2.2. Persulfate oxidation (sulfate radical-based oxidation)

In this study sulfate radicals are created in water using persulfate. Sulfate radicals can be created by several methods. One of them is activation by heat. The formation of SO_4_^•–^ with activation by heat is as follows (Eq. (6)). 

**Figure 11:**
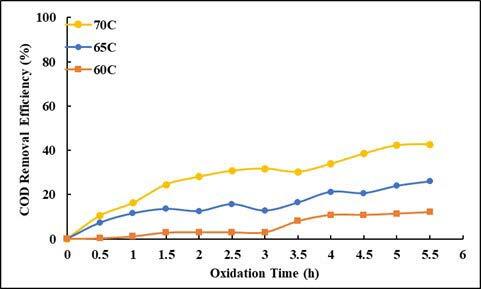
Effect of oxidation time and temperature on COD removal efficiencies (COD/S2O8 = 1/2).

**Figure 12:**
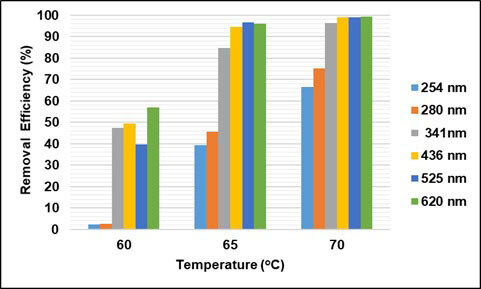
Effect of temperature on UV-VIS absorbance values removal efficiencies (t = 5.5 h)

**Figure 13:**
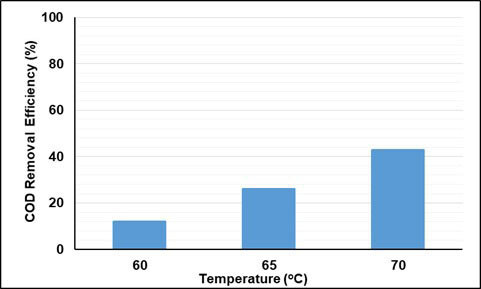
Effect of temperature on COD removal efficiencies (COD/S2O8 = 1/2).

By reacting with several organic compounds, SO_4_^•–^ is more effective than OH^•^, because it is more selective for oxidation, whereas OH^•^ can react rapidly with hydrogen abstraction [26]. Also, sulfate radicals have higher oxidation potentials (2.5 to 3.1 V) than hydroxyl radicals (1.9–2.85 V). In this study, the effect of temperature, time, and COD/S_2_O_8_ were investigated for removal of COD and UV-vis absorbance values in oxidation with persulfate. Here, no studies on the effect of pH have been conducted, and the natural pH of wastewater has been studied. In order to determine the effect of temperature, experiments were carried out at the ratio of COD/S_2_O_8_ = 1/2, at 5.5 h contact time, and at 60, 65, and 70 °C. The effect of temperature and contact time on UV-vis absorbance values and COD removal efficiencies are given in Figures 11–13. As can be seen from the figures, removal efficiencies of COD and UV-vis absorbance values increase with increasing temperature. It is seen that the removal efficiency reaches equilibrium when the contact time is 5.5 h. With increasing temperature from 60 °C to 70 °C, COD removal efficiency increased from 12% to 42%. With increasing temperature, the absorbance values removal efficiencies at 254 and 280 nm wavelengths increased from 2% to 67%, and from 3% to 75% respectively. The absorbance removal efficiency at 341 nm wavelength increased from 47% to 96%. The removal efficiency of absorbance values at wavelengths of 436, 525, 620 nm has increased from 39% to 99%.

**Figure 14:**
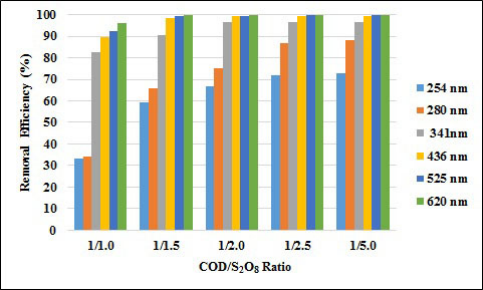
Effect of COD/S2O8 ratio on UV-vis absorbance values removal efficiencies (T: 70  C, t: 5.5 h).

**Figure 15:**
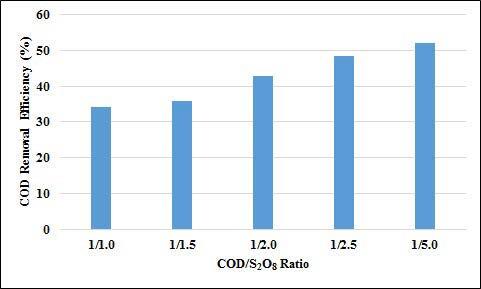
Effect of COD/S2O8 ratio on COD removal efficiencies (T:70 oC, t: 5.5 h).

In the study, the COD/S_2_O_8_ ratio was changed between 1/1 and 1/5 at 70 °C and 5.5 h contact time. The effect of COD/S_2_O_8_ ratio on COD and UV-vis absorbance values removal efficiencies are given in Figures 14 and 15. As can be seen from the figures, removal efficiencies of UV-vis absorbance values and COD removal efficiencies increased as the COD/S_2_O_8_ rate increased. The COD removal efficiency increased from 34% to 52% when the rate was changed from 1/1 to 1/5. The removal efficiency of the absorbance values of UV 254 and 280 nm wavelengths has increased from 1/1 to 1/5, from 33% to 73%, and 34% to 88%, respectively. The absorbance removal efficiencies at UV 436, 525, and 620 nm increased from 89% to 99%. According to these results, it can be said that chromophore groups can be easily broken down by oxidation and therefore their removal efficiency is so high.

When the results are examined, it is seen that especially the color showing the absorbance values of 400–700 nm is removed with high efficiency by oxidation. The oxidation process with sulfate radicals indicates that the chromophore groups, which are the source of the true color, have high removal efficiencies by sulfate radical-based oxidation.

### 3.3. Ozone oxidation

**Figure 16:**
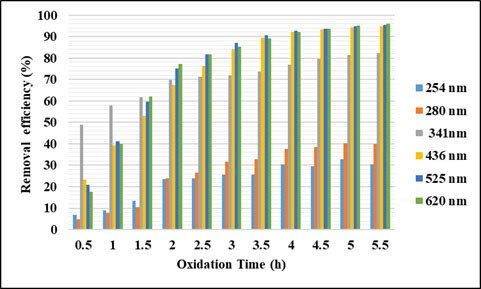
Effect of ozonation on UV-vis absorbance values removal efficiencies.

**Figure 17:**
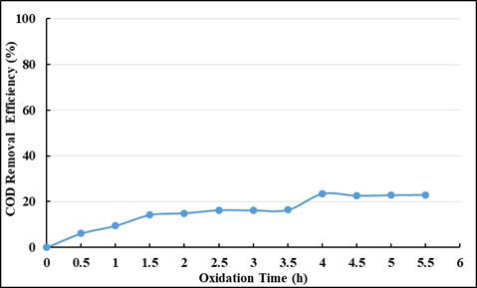
Effect of ozonation on COD removal efficiencies.

In order to compare the sulfate radical-based oxidation and ozonation process, ozone was applied at the natural pH of the wastewater and at a contact time of 5.5 h. After the ozonation, UV-vis absorbance values and COD removal efficiencies are given in Figures 16 and 17. When the removal efficiencies of UV 254 and UV 280 nm wavelengths are examined, it is seen that the removal efficiencies reaching 30% and 39% respectively, at 5.5 h contact time. The removal efficiencies at UV 341 nm wavelength reached to equilibrium and removal efficiency was 83%. The removal efficiency of visible light absorbance values, which is the indicator of true color, is above 95% after 5.5 h. When COD removal efficiencies are analyzed, low removal efficiencies were encountered. As can be seen from Figure 17, the highest COD removal efficiency after ozonation of 5.5 h was 23%. 

It is seen that the absorbance removal efficiencies at UV 254 and 280 nm absorbance values and COD removal efficiencies showed parallel results with the ozonation process and were removed with low efficiencies.

### 3.4. Comparison of the treatment processes

In this study, removal efficiencies of COD and the removal efficiencies of absorbance values of UV-vis at 254, 280, 341, 436, 525, and 620 nm wavelengths were tried to be determined by using adsorption, Fenton, persulfate oxidation, and ozone oxidation in wastewater which has been pretreated by coagulation and flocculation process. 

When Table 2 was examined, it was seen that the color was removed with high efficiencies in all processes, whereas the UV-vis absorbance values representing the organics and aromaticity were removed with lower efficiencies. Chromophore groups are groups that give visible color and in this study, 3 different wavelengths, i.e. 436, 525, 620 nm, were used to measure this value. In all processes, it was observed that the true color was removed with efficiencies over 90%. Especially in the adsorption process, it is seen that the removal efficiencies were above 98% in all wavelengths between 254 and 620 nm. The most important reason for this is that most of the dyes and organics have high adsorption ability on adsorbents under suitable conditions. Adsorption efficiencies were considered to be high because the adsorbent has a positive charge below the pHpzc and the organics are negatively charged due to dissolution at pH: 5. It is seen that COD removal efficiency in adsorption process was higher than other processes and it was 77.5%. 

**Table 2:** Comparison of the adsorption method, oxidation method and AOPs.

Parameters	Adsorption removal efficiency (%)	Ozonation removal efficiency (%)	Advanced oxidation processes removal efficiency (%)	
			Fenton	Persulfate oxidation
COD	77.5	22.9	55.6	52.2
254 nm	98.4	30.3	65.8	72.9
280 nm	99.1	39.9	60.1	88.2
341 nm	99.1	82.4	83.1	96.5
436 nm	99.5	94.8	91.2	99.6
525 nm	99.6	95.5	89.9	99.7
620 nm	99.7	96.1	92.2	99.9

As it is known, Fenton process is a process that includes oxidation, coagulation, and precipitation together. The process examined differs from the other AOPs in terms its removal mechanism. As can be seen from Table 2, in the Fenton process, the absorbance removal efficiencies at the wavelengths of 436, 525, and 620 nm, were around 90%. It is seen that the removal efficiency of the parameters showing the organics and aromaticity was 65% at most. According to a study Fenton oxidation sometimes gives inefficient degradation because of iron-organic complexes [7]. 

When persulfate oxidation and Fenton process were compared, it is seen that persulfate oxidation was more successful in removing dissolved organics (UV280 nm absorbance values), aromaticity (UV254 nm absorbance values), and color (absorbance values at 436, 525, 620 nm). The reason for this is thought to be due to the higher oxidation potential of sulfate radicals (2.5 to 3.1 V) than hydroxyl radicals (1.9 to 2.85 V). Although the ozonation process was not efficient in removing organic materials, it is seen that the removal efficiencies of absorbance value of 341 nm and color (436, 525, 620 nm absorbance values) were high. According to these results, the ozonation process was not sufficient to remove aromatics, but it seems to be suitable for the removal of chromophore groups (color).

## 4. Discussion

In this study, adsorption process, ozonation, and advanced oxidation processes were used for COD and UV 254–280 nm (aromaticity and organics), UV 341 nm (which maximum absorbance is measured), visible light at 43, 525, 620 nm (true color) in wastewater of a chemical producing industry which is pretreated by coagulation-flocculation by FeCl_3_. Fenton at pH 3, persulfate oxidation and ozonation at natural pH were used as AOPs. In the study, the efficiency of COD, aromaticity, and color removals from such complex industrial wastewater has been tried to be determine by adsorption, oxidation and AOPs. In the results of the study, interesting results were obtained about how COD, aromaticity and color removal in such this real wastewater are affected by the mentioned processes. The results and discussion of the study are summarized below:

· The best removal efficiencies in terms of COD, aromaticity, and color were achieved by adsorption. The reason for this is the high interest of organics and dyes to adsorbent at appropriate pH. The most suitable pH was determined as 5. The pH_pzc_ of the activated carbon used as adsorbent was determined as 7.45. Adsorbent is positively charged at pH 5. At this pH, it is thought that the organics, aromatics, and chromophore groups in the wastewater are negatively charged; therefore, their removal efficiencies were high. Although the efficiency of the adsorption process is high, additional studies should be carried out on the required frequency of the regeneration process, the removal of secondary waste and the cost of all these.

· The Fenton process is a process that includes oxidation, coagulation, and sedimentation. When the Fenton results are examined, it is seen that the color removal was around 90%, and the removal efficiency of aromaticity and other UV-vis absorbance values is 65% and 60% respectively. It is seen that the efficiency of COD removal remains at 55%. The most important reason for this is thought to be the formation of iron-organic complexes with Fenton oxidation. According to some studies, Fenton oxidation sometimes gives inefficient degradation due to iron-organic complexes [7]. 

· When ozone and persulfate oxidation is examined, it is seen that the removal efficiencies of organic substances (COD and UV280 nm absorbance values) and aromaticity (UV254 nm absorbance values) by ozonation were quite low compared to persulfate oxidation. The most favorable conditions in persulfate oxidation were a temperature of 70 °C, 5.5 h, and COD/S_2_O_8_ ratio of 1/5. Under these conditions, COD removal efficiency was found at 52%, and the absorbance efficiency of UV254 nm and UV280 nm was 73% and 88%, respectively. UV 341 nm absorbance removal efficiency was above 96.5% and color removal efficiency was above 99%.

· When the ozonation results were examined, it was observed that the removal efficiencies of COD, aromaticity, and natural organic substances remained quite low. The color removal efficiency was found to be around 95% and quite high. Accordingly, ozonation was sufficient to break the bonds of chromophore groups but could not provide the break the bonds of the organics. In this study, ozonation was done at the natural pH (pH: 8) of the wastewater. At this pH, hydroxyl radicals are not formed by ozonation, only ozone oxidation takes place. This is thought to be the reason why these removal efficiencies were low for organics. Although the removal efficiency of the ozone process remained low, it should be investigated whether the removal efficiency will increase as the pH value of the wastewater and the applied ozone dose increase.

· One of the important results of this study in this wastewater, which has been pretreated by coagulation-flocculation, is that the color can be removed more easily than aromatic organics and dissolved organics using the oxidation process. According to these results, it can be said that chromophore groups can be easily broken down by oxidation.

· When the results are examined, it is seen that both adsorption and Fenton processes provide the required 1000 mg/L COD value for discharge to the central wastewater treatment plant. In this case, the adsorption process can be considered primarily due to its higher removal efficiency and ease of operation.
